# Up-Regulation of SH3TC2 Induced by YTHDF1 Predicts Poor Outcome and Facilitates Cell-Cycle Progress in Colorectal Cancer

**DOI:** 10.1155/2022/1600611

**Published:** 2022-12-15

**Authors:** Huili Wu, Feifei Chu, Lu Li, Kunkun Li, Xingguo Xiao, Li Zhang, Yong Zhang

**Affiliations:** ^1^Department of Gastroenterology, Zhengzhou Central Hospital Affiliated to Zhengzhou University, Zhengzhou 450007, China; ^2^Medical Key Laboratory of Diagnosis and Treatment of Colorectal Cancer in Henan Province, Zhengzhou 450007, China; ^3^Zhengzhou Key Laboratory of Diagnosis, Treatment and Research of Colorectal Cancer, Zhengzhou 450007, China; ^4^Branch Center of Advanced Medical Research Center, Zhengzhou Central Hospital Affiliated to Zhengzhou University, Zhengzhou 450007, China

## Abstract

N6-methyladenosine (m6A) modification plays a crucial role in determining the fate and function of RNA transcripts in tumor cells. Nevertheless, how m6A regulates the expression of key molecules and coordinates its involvement in the development of colorectal cancer (CRC) remains largely unclear. Here, we report that the m6A reading protein YTHDF1-mediated up-regulation of SH3TC2 promotes CRC growth both in vitro and in vivo. In a pan-cancer analysis across more than thirty types of cancer, we found that SH3TC2 was dysregulated in nine cancers, including BLCA, CHOL, COAD, LAML, PAAD, READ, SKCM, BRCA, and TGCT, and was closely associated with patient prognosis in four cancers, including COAD, MESO, PAAD, and READ. In particular, SH3TC2 was overexpressed in CRC as confirmed by six independent study cohorts. Clinically, high expression of SH3TC2 predicted worse disease-free survival (DFS) in CRC patients. SH3TC2 showed fascinating diagnostic value and was correlated with immunosuppression in CRC. Functionally, RNA-sequencing combined with experiments revealed that knockdown of SH3TC3 significantly inhibited cell-cycle progress of CRC, impairing cell growth. Mechanistically, YTHDF1 protein directly binds with SH3TC2 mRNA and promotes its elevation in an m6A-dependent manner. Thus, our findings provide a mechanism to target the YTHDF1/SH3TC2 axis for CRC therapy.

## 1. Introduction

Colorectal cancer (CRC) is one of the most prevalent malignant tumors of the digestive system worldwide, which poses a serious challenge to human health. Sung and colleagues published the Global Cancer Statistics 2020, which reported that CRC ranked among the top three out of 36 human cancers in terms of morbidity and mortality [1]. Over the past decade, the number of CRC-related deaths worldwide has increased from 600,000 in 2008 to 900,000 in 2020 [1, 2]. The survival of CRC patients has been prolonged as a result of improved early screening techniques and clinical treatment strategies [3, 4]. However, tumor recurrence and progression mediated by unknown mechanisms remain major factors affecting clinical outcomes in CRC patients [5, 6]. Therefore, it is urgent to explore the molecular mechanism driving the occurrence and development of CRC.

RNA m6A modification is a common form of epigenetic modification that occurs in eukaryotic cells. Under normal conditions, methyltransferase-mediated methylation and demethylase-mediated demethylation are in relative balance, and once the intracellular m6A modification state is out of balance, the biological function of the cell is also changed [7, 8]. Recent studies have shown that tumor cells require m6A modification to maintain their malignant growth, progression, and therapeutic resistance [9, 10]. As one of the m6A reading proteins, YTHDF1 determines the fate of m6A-labeled RNA transcripts, affecting their stability, localization, or translation efficiency [11, 12]. However, how YTHDF1 regulates the expression of a key molecule in CRC has not been well characterized.

SH3TC2, located on chromosome 5 with a 26,468 bp transcript, was initially identified as a gene closely associated with neurodevelopmental abnormalities in humans [13, 14]. But the clinical relevance and biological function of SH3TC2 in human cancer remain largely unclear. In this study, we for the first time delineated the expression and prognostic landscapes of SH3TC2 in more than 30 cancers. SH3TC2 was shown to be abnormally expressed and significantly associated with disease-free survival (DFS) in many cancers, especially CRC. We then focused on the biological role of SH3TC2 in CRC in this work later. Functionally, the deletion of SH3TC2 significantly attenuated the growth ability of CRC in vitro and in vivo. Mechanistically, the m6A recognition protein YTHDF1 binds to and induces up-regulation of SH3TC2 in CRC. Thus, our study advances the understanding of SH3TC2's role in pan-cancer and provides a reference for developing SH3TC2 as a clinical biomarker and/or target for CRC.

## 2. Materials and Methods

### 2.1. GEPIA Database

GEPIA is a comprehensive tumor database containing gene expression and prognostic information for more than thirty common human cancers [15]. In this study, the database was used to analyze the expression of related genes in tumor and normal samples, the relationship with DFS of tumor patients, and the expression correlation between genes.

### 2.2. TISIDB Database

TISIDB is an integrated database for customers to analyze the interactions between tumors and the immune system [16]. It provides a valuable resource for users to explore the immunotherapy of tumors. In this study, we used this database to investigate the relationship between SH3TC2 expression and tumor immune cell infiltration in pan-cancer.

### 2.3. Gene Expression Omnibus (GEO) Datasets

SH3TC2 expression data in four independent CRC cohorts, GSE9348 (12N vs. 32T), GSE32323 (17N vs. 17T), GSE21510 (25N vs. 123T), and GSE39582 (19N vs. 566T), were obtained from the GEO database. According to the expression level of SH3TC2 in tumor and normal samples, the receiver operating characteristic (ROC) curve was used to analyze its diagnostic value in each GEO dataset.

### 2.4. CRC Tissue Specimens and Immunohistochemistry (IHC)

Two independent cohorts were used for validating the expression of SH3TC2 in CRC. Cohort one included paraffin-embedded tissue samples from 12 CRC patients for IHC staining that were collected from Zhengzhou Central Hospital Affiliated to Zhengzhou University between 2018 and 2020 (ZZCH cohort), and this study was approved by the Medical Ethics Committee. Cohort two was a tissue microarray (TMA) containing 30 pairs of CRC samples purchased from Alenabio Company (Xi'an, China) for IHC test (TMA cohort). IHC analysis of paraffin-embedded tissues was supported by Servicebio Company (Wuhan, China). The primary antibodies used in this study were anti-SH3TC2 (Abcam, USA).

### 2.5. Cell Culture and Transfection

Two human CRC cell lines, HCT116 and SW480, were obtained from Procell (Wuhan, China) and the National Infrastructure of Cell Line Resource (NICR), respectively. HCT116 and SW480 cells were cultured in McCoy 5A (Procell, China) and IMDM (Procell, China) medium containing 10% FBS, respectively. Cells were maintained in a wet incubator with 5% CO_2_ at 37°C. The recombinant lentivirus used for SH3TC2 knockdown (sh-SH3TC2#1, #2) and the corresponding control lentivirus (sh-NC) were purchased from GeneChem (Shanghai, China). The overexpression plasmids for wide-type (OE-YTHDF1-WT) and mutant (OE-YTHDF1-Mut) YTHDF1 were also obtained from GeneChem (Shanghai, China). The reagents HiTransG A (GeneChem, China) and Lipo3000 (Thermo, USA) were utilized to transfect lentiviruses and plasmids into CRC cells, respectively.

### 2.6. RNA Sequencing

HCT116 cells infected with sh-NC lentivirus or sh-SH3TC2#1 lentivirus were used for transcriptome sequencing (3 replicates per sample). The sequencing process was similarly described in the previous study [17]. Firstly, total RNAs were extracted by the Trizol method, RNA purity was detected by spectrophotometer, and RNA integrity was analyzed by agarose gel electrophoresis and the Agilent 2100 BioAnalyzer. The library was constructed using Illumina's NEBNext® UltraTM RNA Library Prep Kit. Initial quantitative analysis was performed using a Qubit2.0 fluorometer, and the library was diluted to 1.5 ng per *μ*L. Agilent 2100 BioAnalyzer and qRT-PCR were used to detect library quality. Then, the Illumina platform was used for library sequencing, and a 150 bp paired terminal reading was generated to obtain the sequence information of the fragment to be measured. After quality control and sequence alignment based on the reference genome, DESeqv2 software was used to analyze the differentially expressed genes (DEGs) between the two groups. Finally, the DEGs were used for gene enrichment analysis based on gene ontology (GO) and the Kyoto Encyclopedia of Genes and Genomes (KEGG).

### 2.7. Cell Cycle Analysis

Propidium Iodide (PI) staining kit (7Sea, China) and flow cytometer (Beckman, USA) were used to detect the changes of cell cycle in CRC cells with SH3TC2 deletion. In short, the adherent cells were digested with trypsin solution to collect cell deposits. The cells were immobilized with precooled 70% ethanol at 4°C for 3 h. Finally, cells were incubated with PI staining solution at 37°C for half an hour and detected by flow cytometry.

### 2.8. Cell Growth Assays

A colony formation experiment was used to evaluate the in vitro nonpopulation-dependent growth of CRC cells with SH3TC2 deficiency. The cells were seeded in 12-well plates with 800 cells in each well. About 9 to 12 days later, visible cellular colonies appeared in the plates. After fixation and crystal violet staining, the colonies were photographed and analyzed. For the CCK-8 growth assay, the transfected cells were inoculated into 96-well plates, and 10 *μ*L of CCK-8 solution was added into the wells and incubated at 37°C for 3 to 4 hours. Finally, the light absorption value of each well at 450 nm was measured by using a microplate reader.

### 2.9. Animal Experiment

The in vivo growth ability of CRC cells with SH3TC2 ablation was investigated by a subcutaneous tumor-forming experiment in nude mice. HCT116 cells infected with sh-NC lentivirus or sh-SH3TC2 #1 lentivirus were used to inoculate nude mice with 5 mice in each group. Each BALB/c mouse was injected with 5 × 106 cells. When obvious tumors appeared subcutaneously in nude mice, the formula length × width × width × 3.14/6 was used to calculate the tumor volume. Finally, tumors from nude mice were isolated and used for further analysis. The study was reviewed and approved by the Ethics Committee of Zhengzhou Central Hospital Affiliated to Zhengzhou University.

### 2.10. Western Blot

A Western blot was used to detect protein levels of corresponding genes in CRC cells or nude mouse tumor tissues. In brief, total protein was extracted using a lysate mixture containing RIPA and protease inhibitors. After concentration determination, an equal amount of total proteins from each sample was used for polyacrylamide gel electrophoresis. After membrane transfer, blocking, antibody incubation, and coloration, protein bands on PVDF membranes were used for detection and analysis. Primary antibodies used in this study include anti-GAPDH (Bioworld, China), anti-SH3TC2 (Bioss, China), anti-CDK4 (Bioworld, China), anti-Cyclin D1 (Bioworld, China), and anti-YTHDF1 (Abcam, USA).

### 2.11. Quantitative Real-Time Polymerase Chain Reaction (qRT-PCR)

The mRNA expression of SH3TC2 in CRC cells following YTHDF1 silence was detected by qRT-PCR. The Trizol method was utilized to extract total RNAs from CRC cells. After concentration and purity determination, total RNAs were used to synthesize cDNAs. Then, the SYBR Green mix kit (DBI, Germany) and 7500 Fast system were utilized for qRT-PCR detection. The housekeeping gene GAPDH was used as an internal reference, and gene expression wasanalyzed by the 2−ΔΔ*Ct* method. Primers are shown in Supplementary Table 1.

### 2.12. RNA m6A Prediction of SH3TC2

The RMBase v2.0 online server (https://rna.sysu.edu.cn/rmbase/) based on epitranscriptome sequencing data was employed to predict the m6A modification on SH3TC2 mRNA. The m6A reading protein that may bind with SH3TC2 was analyzed by the m6A2Target algorithm (http://m6a2target.canceromics.org/).

### 2.13. Methylated RNA Immunoprecipitation (MeRIP) Analysis

The m6A modifications on SH3TC2 mRNA were detected by using the MeRIP kit (Millipore, USA). First, the Trizol method was used to extract total RNAs from CRC cells, and the RNA concentration was adjusted to 1 *μ*g per *μ*L for each sample. The RNA was then segmented with a crushing buffer and incubated with magnetic beads coupled with IgG or m6A antibodies at 4°C for 3 h. Finally, PCR and agarose gel electrophoresis were used to detect m6A-labeled SH3TC2 transcripts. The primer for MeRIP-PCR is displayed in Supplementary Table 1.

### 2.14. RNA-Protein Immunoprecipitation (RIP) Assay

The direct interaction between YTHDF1 protein and SH3TC2 mRNA was detected by the RIP kit (Millipore, USA). In summary, CRC cells were cleaved using a mixture of buffer containing RNA inhibitors and protease inhibitors. Cell lysates and magnetic beads coupled with IgG or YTHDF1 antibodies were then incubated in immunoprecipitation buffer at 4°C for 2 to 4 h. Finally, the immunoprecipitated samples containing RNA were purified and analyzed by PCR and agarose gel electrophoresis.

### 2.15. Statistical Analysis

The student's *t*-test was used to compare the data differences between any two groups, and a *p* value less than 0.05 was considered statistically significant. The Kaplan–Meier method was utilized for patient survival analysis. SPSS 19.0 and GraphPad 9.0 were used for statistical analysis and data graphing.

## 3. Results

### 3.1. The Expression Panorama of SH3TC2 in Pan-Cancer across 31 Human Cancers

**Figure 1:**
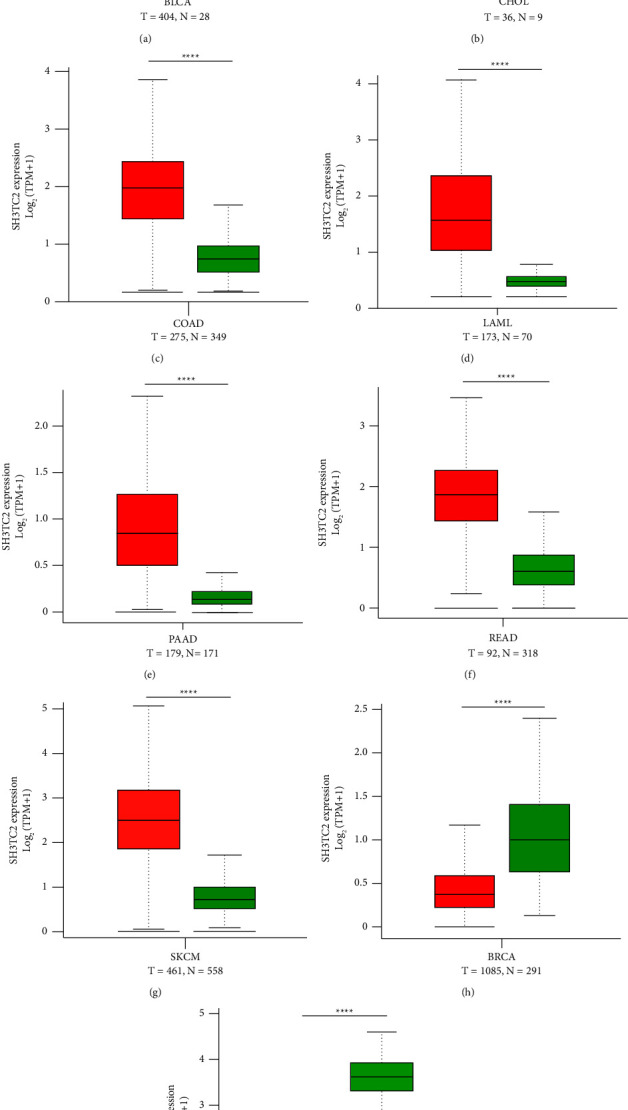
Pan-cancer analysis based on the GEPIA database reveals that SH3TC2 is dysregulated in 9 human cancer types. (a) SH3TC2 was upregulated in 404 BLCA samples compared with 28 normal samples, ^*∗*^*p* < 0.05. T represents tumor, and N represents normal. (b) SH3TC2 was increased in 36 CHOL samples compared with 9 normal samples, ^*∗∗*^*p* < 0.01. (c) SH3TC2 was elevated in 275 COAD samples compared with 349 normal samples, ^*∗∗∗∗*^*p* < 0.0001. (d) SH3TC2 was overexpressed in 173 LAML specimens compared with 70 normal specimens, ^*∗∗∗∗*^*p* < 0.0001. (e) SH3TC2 was highly expressed in 179 PAAD specimens compared with 171 normal specimens, ^*∗∗∗∗*^*p* < 0.0001. (f) SH3TC2 was upregulated in 92 READ specimens compared with 318 normal specimens, ^*∗∗∗∗*^*p* < 0.0001. (g) SH3TC2 was elevated in 461 SKCM tissues compared with 558 normal tissues, ^*∗∗∗∗*^*p* < 0.0001. (h) SH3TC2 was decreased in 1085 BRCA tissues compared with 291 normal tissues, ^*∗∗∗∗*^*p* < 0.0001. (i) SH3TC2 was downregulated in 137 TGCT tissues compared with 165 normal tissues, ^*∗∗∗∗*^*p* < 0.0001.

To explore the potential role of SH3TC2 in human cancer, we for the first time analyzed the expression landscape of SH3TC2 in 31 cancer types by interrogating the comprehensive cancer database GEPIA. For this, we defined a fold change greater than 1.5 and *p* value less than 0.05 as a significant difference in SH3TC2 expression between tumor and normal samples in each cancer type. We found that SH3TC2 was significantly overexpressed in 7 cancers, including bladder urothelial cancer (BLCA), cholangio cancer (CHOL), colon adenocarcinoma (COAD), acute myeloid leukemia (LAML), pancreatic adenocarcinoma (PAAD), rectum adenocarcinoma (READ), and skin cutaneous melanoma (SKCM), while downregulated in 2 cancers, breast invasive carcinoma (BRCA), and testicular germ cell tumors (TGCT), compared with the corresponding normal tissue samples (Figures 1(a)–1(i)). However, there was no significant difference in SH3TC2 expression levels between normal and tumor samples of the other 22 cancers (Supplementary Figures 1A–1C). These results suggest that SH3TC2 may play a carcinogenic role in some specific cancers, such as BLCA, CHOL, etc.

### 3.2. The Prognostic Landscape of SH3TC2 in Human Pan-Cancer

**Figure 2:**
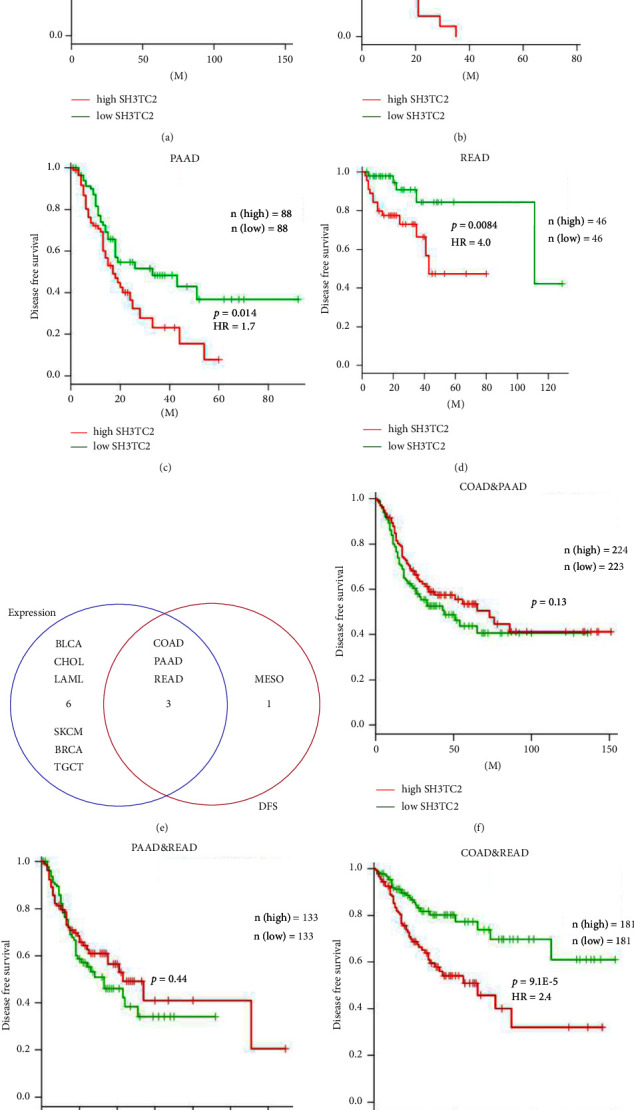
Survival analysis based on the GEPIA database uncovers that SH3TC2 is associated with patient prognosis in 4 human cancer types. (a) High expression of SH3TC2 was associated with poor patient DFS in COAD, *p*=0.0015, HR = 2.2, *n* (high) = 135, and *n* (low) = 135. (b) High level of SH3TC2 predicted worse patient DFS in mesothelioma (MESO), *p*=0.0062, HR = 2.3, *n* (high) = 41, and *n* (low) = 38. (c) High SH3TC2 expression indicated undesirable patient DFS in PAAD, *p*=0.014, HR = 1.7, *n* (high) = 88, and *n* (low) = 88. (d) SH3TC2 was a risk factor for unfavorable patient DFS in READ, *p*=0.0084, HR = 4.0, *n* (high) = 46, and *n* (low) = 46. (e) Crossanalysis showed that SH3TC2 was abnormally expressed in 3 cancers (COAD, PAAD, and READ) and significantly correlated with DFS of patients. (f) The relationship between SH3TC2 expression and patient DFS in COAD and PAAD, *p*=0.13, *n* (high) = 224, and *n* (low) = 223. (g) The relationship between SH3TC2 expression and patient DFS in PAAD and READ, *p*=0.44, *n* (high) = 133, and *n* (low) = 133. (h) High SH3TC2 expression indicated worse patient DFS in COAD and READ, *p* = 9.1*E* − 5, HR = 2.4, *n* (high) = 181, and *n* (low) = 181.

**Figure 3:**
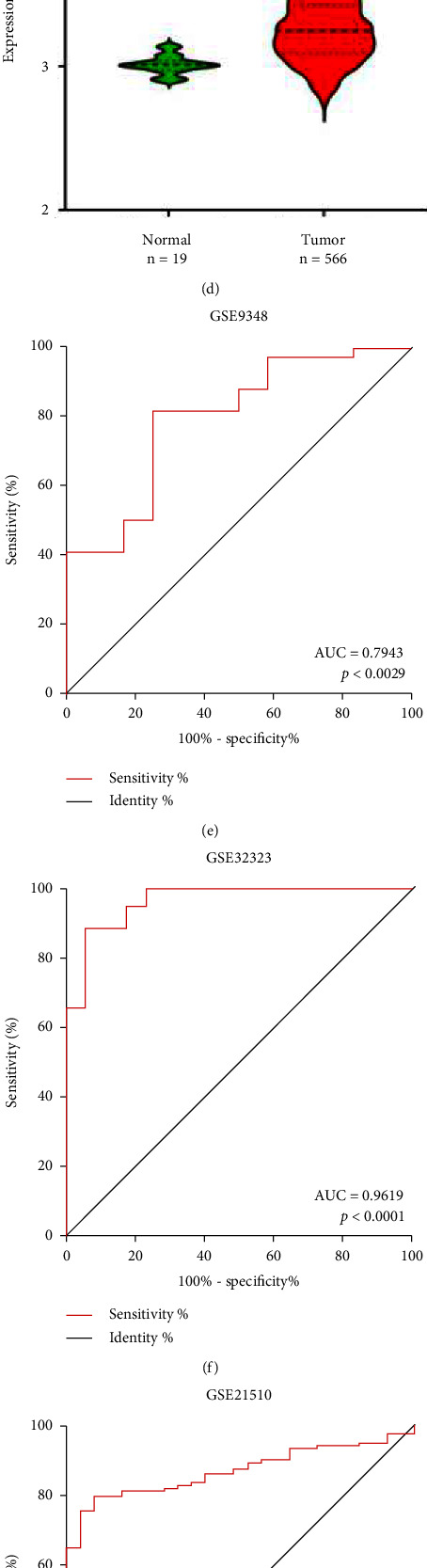
SH3TC2 serves as a potential diagnostic biomarker in CRC. (a) The expression of SH3TC2 in 32 CRC tumor tissues and 12 normal tissues was analyzed from GSE9348, ^*∗∗∗*^*p* < 0.001. (b) The expression of SH3TC2 in 17 pairs of CRC tumor and normal tissues was analyzed from GSE32323, ^*∗∗∗*^*p* < 0.001. (c) The expression of SH3TC2 in 123 CRC tumor tissues and 25 normal tissues was analyzed from GSE21510, ^*∗∗∗∗*^*p* < 0.0001. (d) The expression of SH3TC2 in 566 CRC tumor tissues and 19 normal tissues was analyzed from GSE39582, ^*∗∗∗∗*^*p* < 0.0001. (e-f) The diagnostic value of SH3TC2 in four independent CRC study cohorts, GSE9348 (e), GSE32323 (f), GSE21510 (g), and GSE39582 (h), was analyzed using the ROC curve. AUC refers to the area under the curve, and the higher the AUC value, the higher the diagnostic value. (i) The expression of SH3TC2 in 12 pairs of CRC tissues was tested by IHC, ^*∗*^*p* < 0.05. (j) The expression of SH3TC2 in 30 pairs of CRC tissues from the TMA cohort was examined by IHC, ^*∗∗*^*p* < 0.01.

Given that SH3TC2 is dysregulated in some cancers, we wondered whether SH3TC2 expression could predict patient clinical outcomes. To this end, we used GEPIA database to comprehensively analyze the prognostic significance of SH3TC2 in 33 human cancers, that is, the relationship between SH3TC2 expression and DFS of cancer patients. Patients with each cancer type were divided into high and low expression groups based on the median value of SH3TC2 expression. Results showed that SH3TC2 expression was independently associated with DFS in four types of cancer, including COAD (Figure 2), mesothelioma (MESO) (Figure 2(b)), PAAD (Figure 2(c)), and READ (Figure 2(d)). High expression of SH3TC2 predicts shorter DFS in patients. Next, we cross-analyzed the expression (Figure 1) and prognosis (Figures 2(a)–2(d)) of SH3TC2, and found that SH3TC2 was dysregulated in three cancers and significantly correlated with poor DFS in patients; they are COAD, PAAD, and READ (Figure 2(e)). We then wondered whether SH3TC2 expression could predict clinical outcomes in any two types of cancer among COAD, PAAD, and READ. SH3TC2 showed a more significant prognostic association only when COAD patients and READ patients were included in an integrated analysis (Figures 2(f)–2(h)). Since both COAD and READ belong to colorectal cancer (CRC) in origin, these results strongly suggest that SH3TC2 may play a key role in the development of CRC (See Figure3).

### 3.3. SH3TC2 Correlates with Tumor Immunity

Emerging studies have shown that infiltrating immune cells in the tumor microenvironment affect tumor progression and therapeutic effect [18, 19], and lymphocyte infiltration may be used as a prognostic marker of CRC [20]. We are interested to know whether SH3TC2 has some correlation with tumor immune status. Therefore, we used the TISIDB database to comprehensively observe the relationship between SH3TC2 expression and immune cell infiltration in pan-cancer. The results showed that SH3TC2 expression was associated with immune activation in ACC, LUAD, LIHC, etc. and immunosuppression in COAD, READ, etc. (Supplementary Figure 2A). Tumor cells can achieve immune escape by regulating the expression of immune checkpoint molecules (ICMs), and blocking ICMs can enhance the effect of immunotherapy and benefit cancer patients [21, 22]. We then interrogated the GEPIA database to analyze the expression correlation between SH3TC2 and a series of well-known ICMs in the CRC. We found that SH3TC2 was significantly correlated with VTCN1, but not with CTLA4, PDCD1, CD28, or CD70 (Supplementary Figures 2B–2F), implying that SH3TC2 may be involved in CRC immunity through VTCN1.

### 3.4. SH3TC2 Serves as a Potential Diagnostic Biomarker in CRC

Considering that SH3TC2 showed an obviously high expression status in CRC (COAD and READ, Figures 1(c) and 1(f)), we further included four CRC cohorts (GSE9348, GSE32323, GSE21510, and GSE39582) from the GEO database to verify SH3TC2 expression. All these four independent datasets showed that SH3TC2 expression was upregulated in CRC tumor samples (Figures 3(a) and 3(d)). Next, we determined the diagnostic value of SH3TC2 in these four CRC cohorts by using the receiver operating characteristic (ROC) curve and indicating the area under the curve (AUC). Consistently, the four GEO datasets showed that SH3TC2 had a high diagnostic value in CRC, with AUC values ranging from 0.79 to 0.96 (Figures 3(e)–3(h)), suggesting that SH3TC2 is a potential clinical diagnostic marker of CRC. Subsequently, we detected protein levels of SH3TC2 in two additional CRC cohorts using IHC, and SH3TC2 expression was again elevated in CRC tissues compared to normal tissues (Figures 3(i)–3(j)). Collectively, the above findings hint that the up-regulated SH3TC2 may play a role in CRC biology.

### 3.5. RNA Sequencing and Flow Cytometry Reveal the Participation of SH3TC2 in CRC Cell-Cycle Regulation

**Figure 4:**
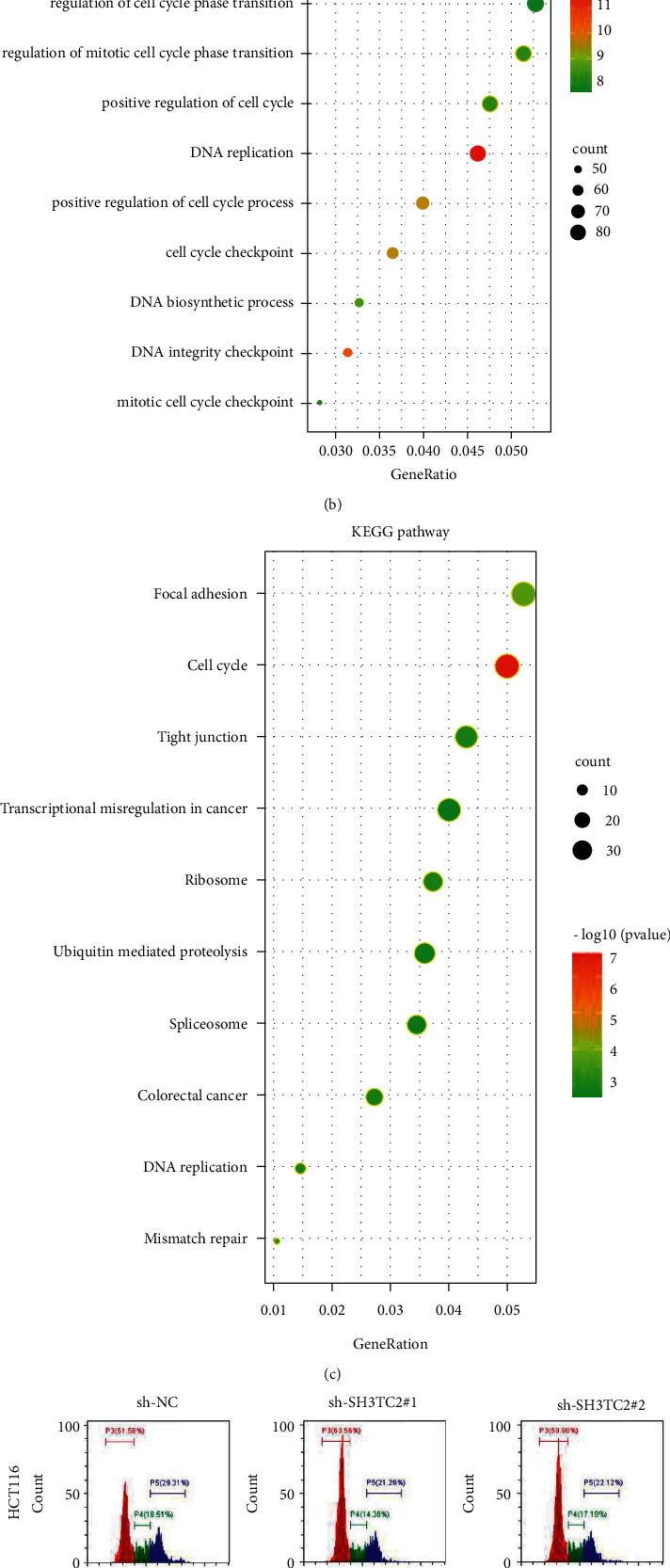
SH3TC2 promotes cell-cycle progress in CRC. (a) RNA sequencing was performed to investigate the molecular expression changes caused by SH3TC2 knockdown in HCT116 cells. NC refers to HCT116 cells transfected with sh-NC lentivirus, and KD refers to HCT116 cells transfected with sh-SH3TC2#1 lentivirus. (b) Gene enrichment analysis of GO (b) and KEGG (c) was performed based on the RNA sequencing result to explore biological pathways related to SH3TC2. (d) Flow cytometry was utilized to detect cell-cycle changes in HCT116 and SW480 cells following SH3TC2 knockdown.

To clarify the biological role of SH3TC2 in CRC, RNA sequencing was performed to observe the molecular expression changes in HCT116 cells after SH3TC2 deletion. We found that knockdown of SH3TC2 caused 868 genes to be downregulated and 790 genes to be up-regulated (Figure 4(a)). We then performed pathway enrichment analysis based on GO and KEGG for these 868 down-regulated genes. GO results showed that SH3TC2-associated genes were mainly enriched in DNA replication, cell cycle checkpoints, positive regulation of cell cycle processes, mitotic cell cycle checkpoints, etc. (Figure 4(b)). KEGG analysis depicted that SH3TC2 may participate in the cell cycle, colorectal cancer, DNA replication, mismatch repair, etc. (Figure 4(c)). Both GO and KEGG suggest that SH3TC2 may be involved in the regulation of the cell cycle in CRC. Subsequently, flow cytometry confirmed that deletion of SH3TC2 could arrest the cycle of HCT116 and SW480 cells at the G0/G1 phase (Figure 4(d)), indicating that SH3TC2 is required for maintaining cell-cycle progress in CRC.

### 3.6. SH3TC2 Promotes CRC Growth Both in Vitro and in Vivo

**Figure 5:**
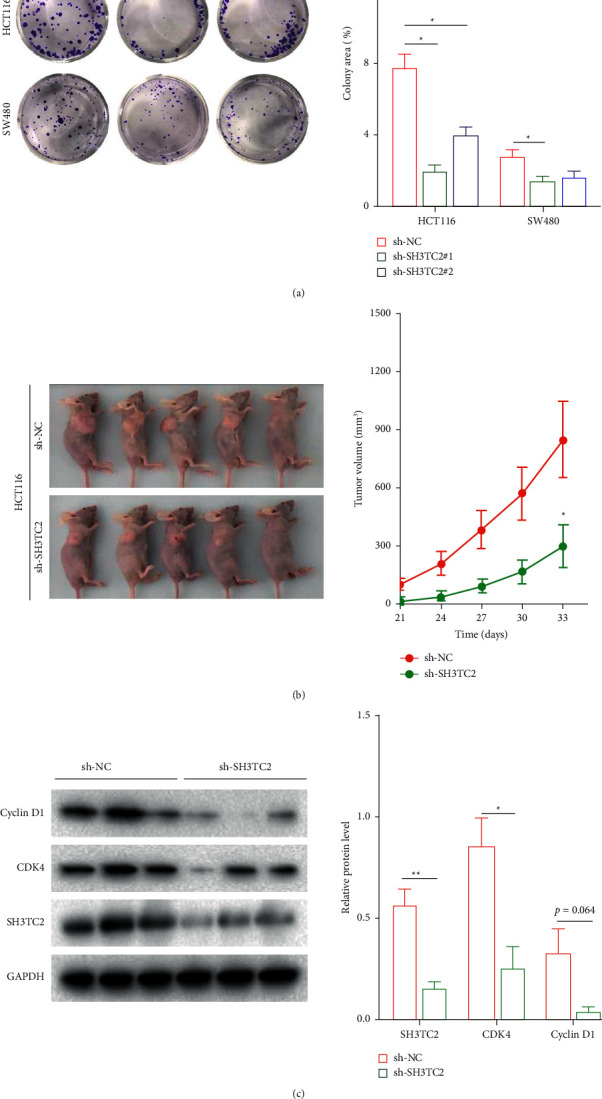
SH3TC2 promotes CRC growth both in vitro and in vivo. (a) The colony formation experiment was used for evaluating the nonpopulation-dependent growth of CRC cells in vitro, ^*∗*^*p* < 0.05. (b) Tumor formation in nude mice indicated that knockdown of SH3TC2 inhibited growth of HCT116 cells in vivo, ^*∗*^*p* < 0.05. (c) The protein levels of SH3TC2, CDK4, and cyclin D1 in tumors were tested by the Western blot assay, ^*∗*^*p* < 0.05 or ^*∗∗*^*p* < 0.01.

Tumor cells maintain their malignant growth through cell-cycle regulation. We next investigated the effects of SH3TC2 deficiency on CRC cell growth. Results from colony formation experiment showed that knockdown of SH3TC2 attenuated the growth of HCT116 and SW480 cells in vitro (Figure 5(a)). In addition, the results of a subcutaneous tumorigenesis experiment in nude mice demonstrated that SH3TC2 knockdown significantly inhibited the growth of HCT116 cells in vivo, presenting a smaller tumor volume (Figure 5(b)). We then detected the protein levels of SH3TC2 and two cell-cycle regulators, CDK4 and Cyclin D1, in the tumor tissues from nude mice by Western blot and found that knockdown of SH3TC2 caused a decrease in CDK4 and Cyclin D1 (Figure 5(c)). These data indicate that ablation of SH3TC2 impairs CRC growth both in vitro and in vivo.

### 3.7. The m6A Reader YTHDF1 Mediates Upregulation of SH3TC2

**Figure 6:**
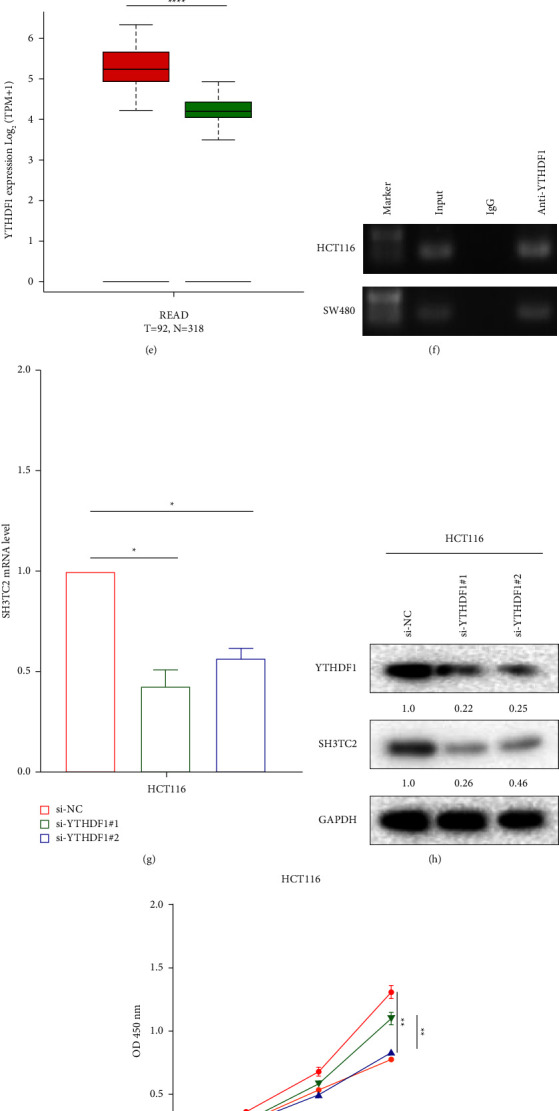
The m6A reader YTHDF1 regulates the expression of SH3TC2 in CRC. (a) The putative m6A modification site on the SH3TC2 transcript was predicted by RMBase v2.0. (b) MeRIP-PCR was employed to detect m6A modification of SH3TC2 mRNA in HCT116 and SW480 cells. (c) The m6A modification of SH3TC2 mRNA in NCM460 and HCT116 cells was detected by MeRIP-PCR. (d-e) The expression of YTHDF1 in both COAD and READ was analyzed by the GEPIA database, ^*∗∗∗∗*^*p* < 0.0001. (f) RIP-PCR was used to detect the direct interaction between YTHDF1 protein and SH3TC2 mRNA in HCT116 and SW480 cells. (g-h) The mRNA and protein levels of SH3TC2 in CRC cells following YTHDF1 knockdown were examined by qRT-PCR (g) and Western blot (h) assays, respectively. (i-j) CCK-8 and flow cytometry assays were used to evaluate cell growth and cell-cycle changes in HCT116 cells.

Considering that SH3TC2 is highly expressed in CRC and is necessary for sustaining CRC growth, we intended to investigate the molecular mechanism that mediates SH3TC2 up-regulation. A number of recent groundbreaking studies have confirmed that m6A epigenetic modification plays a critical role in regulating the stability of intracellular RNA and maintaining RNA pools in cancer cells, which are mainly dependent on the m6A reading protein [23, 24]. In view of this, it is interesting to explore whether the high expression of SH3TC2 in CRC is mediated by m6A modification. First, we used RMBase v2.0 software to predict and obtain a potential m6A modification site at 1669 bp from the 5′-end of SH3TC2 mRNA (Figure 6(a)). We then confirmed the presence of the m6A modification on the SH3TC2 transcript in CRC cells by MeRIP-PCR experiment (Figure 6(b)). Moreover, the m6A enrichment of SH3TC2 transcript in normal colonic epithelial cell NCM460 was lower than that in CRC cells (Figure 6(c)). Next, we used the m6A2Target database to analyze RIP-seq-based data and found that three m6A readers may bind with the SH3TC2 transcript, namely YTHDF1, YTHDF2, and YTHDF3. Subsequently, we used the GEPIA database to investigate the expression levels of these three molecules in CRC and their correlation with the expression of SH3TC2. It was found that both YTHDF1 and YTHDF2, but not YTHDF3, were upregulated in CRC (Figures 6(d) and 6(e), Supplementary Figures 3A–3D). YTHDF1, but not YTHDF2 or YTHDF3, had a significant positive correlation with the expression of SH3TC2 (Supplementary Figures 3E–3G). This suggests that YTHDF1 may play a key role in regulating SH3TC2 expression. Further RIP-PCR analysis confirmed the direct binding of YTHDF1 protein and SH3TC2 transcript in CRC cells (Figure 6(f)). After silencing YTHDF1, both mRNA and protein levels of SH3TC2 were decreased significantly in CRC cells (Figures 6(g) and 6(h)) without affecting the m6A enrichment of SH3TC2 transcript (Supplementary Figure 3H). To further investigate the role of the YTHDF1/SH3TC2 axis in CRC proliferation, we conducted functional rescue experiments. Results showed that overexpression of wild-type YTHDF1 rescued, at least in part, the CRC growth inhibition caused by SH3TC2 knockdown, while transfection of mutant YTHDF1 plasmids did not (Figures 6(i) and 6(j)). Together, these data demonstrate that YTHDF1 regulates the expression of SH3TC2 in CRC and that YTHDF1/SH3TC2 contribute to CRC progression.

## 4. Discussion

Advances in high-throughput sequencing technology in recent decades have greatly improved our understanding of the molecular mechanisms of disease [25, 26]. In the field of molecular oncology, changes in molecular expression between tumor and adjacent normal tissues have been detected by microarray or sequencing technology, and hundreds of DEGs have been identified, accelerating the development of therapeutic targets and diagnostic markers for cancer [27, 28]. Nevertheless, most of the key molecules that drive tumor development have not been fully identified. Here, we report that SH3TC2 is a potential tumor driver and clinical biomarker.

Dysregulation of SH3TC2 expression is considered to be associated with clinical spinal malformation and nervous system structural integrity [13, 14]. Based on in silico analysis, Yu et al. found that increased expression of SH3TC2-DT/SH3TC2 gene pairs was associated with shorter survival in LAML patients [29]. But the specific function ofSH3TC2 in human cancer has not been reported. In order to fully investigate the expression status and clinical prognostic value of SH3TC2 in human cancers, we employed the integrated database GEPIA to analyze the expression profile of SH3TC2 in more than 30 types of cancer and its relationship with patient survival. SH3TC2 was found to be dysregulated in 9 cancers, including BLCA, CHOL, COAD, LAML, PAAD, READ, SKCM, BRCA, and TGCT, and correlated with patient DFS in 4 cancers, including COAD, MESO, PAAD, and READ. These findings suggest that there is significant heterogeneity in the expression of SH3TC2 in different tumors and that SH3TC2 may serve as a therapeutic target or prognostic marker for some specific cancers. In particular, we noted that SH3TC2 expression was significantly reduced in TGCT tumor samples compared to normal samples, and when the threshold was set to 2, SH3TC2 expression was able to distinguish well between 165 normal samples and 137 tumor samples (Figure 1(i)). In future studies, it is possible to develop SH3TC2 as an effective biomarker for early diagnosis of tumors, including but not limited to TGCT, by incorporating large-scale, multicenter clinical blood samples to detect SH3TC2 levels.

The emergence of immunotherapy is regarded as a new hope in the fight against tumors, including CRC. However, inherent or acquired resistance prevents most CRC patients from benefiting from immunotherapy, which is mainly due to the elusive immune escape/suppression mechanisms of tumor [30–32]. By interrogating the TISIDB database, we found that SH3TC2 expression was associated with low levels of immune cell infiltration in CRC, suggesting that SH3TC2 may be involved in the immunosuppression of CRC. VTCN1, also known as B7-H4, a recently discovered ICM, has been found to be associated with immune cell infiltration and poor prognosis for CRC [33, 34]. Our results showed that SH3TC2 was significantly positively correlated with VTCN1 expression in CRC. Although we have not yet elucidated the exact mechanisms by which SH3TC2 participates in the immunosuppression of CRC and the regulation of VTCN1 by SH3TC2, our bioinformatics results provide the first clue to further explore the role of SH3TC2 in CRC immunotherapy.

Corresponding to DNA methylation and histone acetylation, m6A modification provides a new dimension for understanding posttranscriptional regulation of RNA during tumorigenesis [35]. In this study, we demonstrated that the m6A reader protein YTHDF1 directly binds to the SH3TC2 transcript and up-regulates its expression. This explains, at least in part, the molecular mechanism by which SH3TC2 expression is overexpressed in CRC. Recent studies have shown that YTHDF1 plays an important role in chemotherapy resistance and the self-renewal of CRC [36, 37], but the relevant mechanism remains unclear. Our results showed that SH3TC2 promoted the cell-cycle progression of CRC as well as tumor growth and that SH3TC2 was a new downstream target of the YTHDF1 molecule in CRC. In addition, overexpression of YTHDF1 rescued, at least in part, the inhibitory effects of SH3TC2 knockdown on CRC growth and cell-cycle progress. These findings suggest that the YTHDF1/SH3TC2 axis may play a role in the development of CRC, and the regulation network mediated by YTHDF1 needs to be further studied.

In summary, our study reveals for the first time the expression status and prognostic value of SH3TC2 in pan-cancer and demonstrates the critical role of SH3TC2 in maintaining the cell-cycle progress and growth of CRC. Moreover, RNA m6A modification and YTHDF1 binding may be responsible for the up-regulation of SH3TC2 expression in CRC. Our work provides a scientific reference for targeting YTHDF1/SH3TC2 against CRC clinically.

## Data Availability

The datasets supporting our results are available in the public databases GEO and TCGA as well as data sources in the method. The data of our in-house cohort are provided in supplementary tables.

## Supplementary Materials

Supplementary Materials There was one supplemental table and two supplemental figures in the Supplemental File. Supplementary Tables 1. The primers used in this study. Supplementary Figure 1. The expression of SH3TC2 in pan-cancer across 22 human cancers. Supplementary Figure 2. SH3TC2 correlates with tumor immunosuppression in CRC. Supplementary Figure 3. The correlation between SH3TC2 expression and YTHDF1/2/3 in CRC.
