# Supplementary material for: Up-Regulation of SH3TC2 Induced by YTHDF1 Predicts Poor Outcome and Facilitates Cell-Cycle Progress in Colorectal Cancer
Source: J Oncol. 2022 Dec 15;2022:1600611. doi: 10.1155/2022/1600611 (PMC9780001; doi:10.1155/2022/1600611)
Supplement: Supplementary Materials — There was one supplemental table and two supplemental figures in the Supplemental File. Supplementary Tables 1. The primers used in this study. Supplementary Figure 1. The expression of SH3TC2 in pan-cancer across 22 human cancers. Supplementary Figure 2. SH3TC2 correlates with tumor immunosuppression in CRC. Supplementary Figure 3. The correlation between SH3TC2 expression and YTHDF1/2/3 in CRC. [file 1600611.f1.docx]

**Supplementary Tables 1.** The primers used in this study

| Name | Direction | Sequence |
| --- | --- | --- |
| SH3TC2 | Forward | GCCATGGTTCACTGCTACCT |
|  | Reverse | GGGTGGATGATTTGAGCCCA |
| GAPDH | Forward | CCGGGAAACTGTGGCGTGATGG |
|  | Reverse | AGGTGGAGGAGTGGGTGTCGCTGTT |
| MeRIP-PCR | Forward | TCGCCCCCATATTGGCTTTT |
|  | Reverse | TGGATGGCCTCCTCGAAGTA |


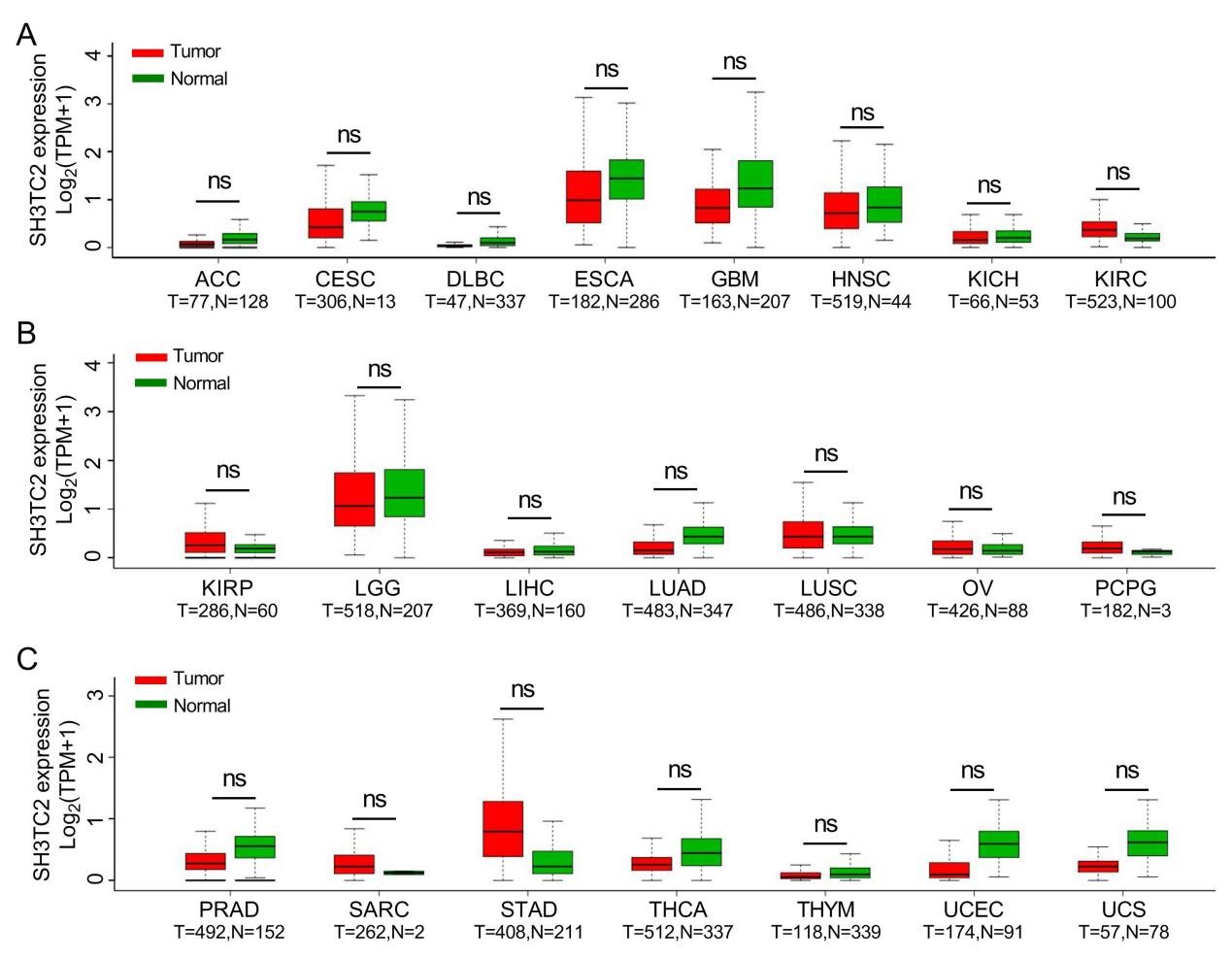


**Supplementary Figure 1. The expression of SH3TC2 in pan-cancer across 22 human cancers**

(A-C) As analyzed by the GEPIA database, SH3TC2 showed no significant difference between tumor and normal tissues in 22 cancer types including adrenocortical carcinoma (ACC), cervical squamous cell carcinoma and endocer vical adenocarcinoma (CESC), lymphoid neoplasm diffuse large B-cell lymphoma (DLBC), esophageal carcinoma (ESCA), glioblastoma multiforme (GBM), head and neck squamous cell carcinoma (HNSC), kidney chromophobe (KICH), kidney renal clear cell carcinoma (KIRC), kidney renal papillary cell carcinoma (KIRP), brain lower grade glioma (LGG), liver hepatocellular carcinoma (LIHC), lung adenocarcinoma (LUAD), Lung squamous cell carcinoma (LUSC), ovarian serous cystadenocarcinoma (OV), pheochromocytoma and paraganglioma (PCPG), prostate adenocarcinoma (PRAD), sarcoma (SARC), stomach adenocarcinoma (STAD), thyroid carcinoma (THCA), thymoma (THYM), uterine corpus endometrial carcinoma (UCEC), and uterine carcinosarcoma (UCS).


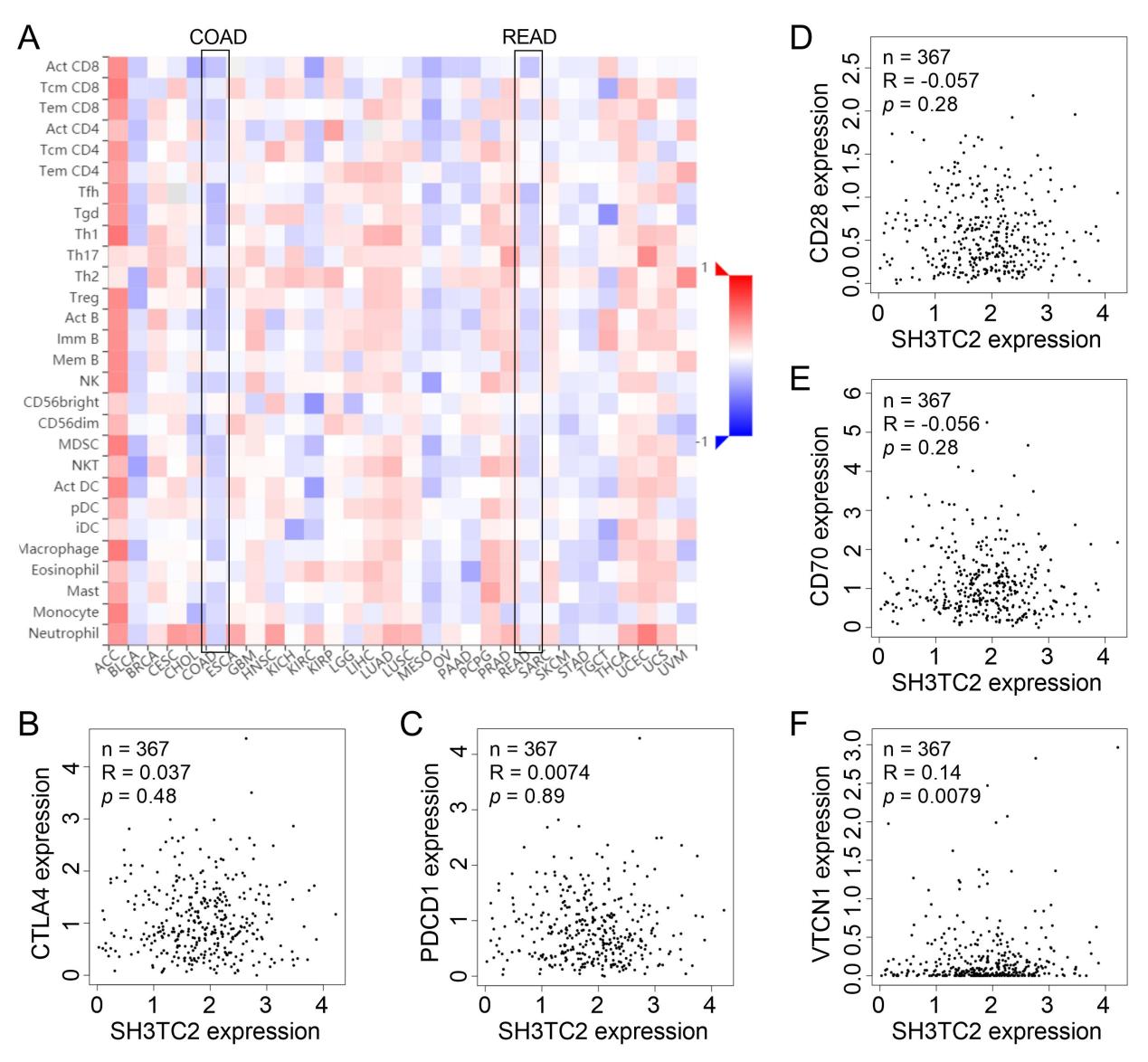


**Supplementary Figure 2. SH3TC2 correlates with tumor immunosuppression in CRC.** (A) The relationship between SH3TC2 expression and tumor immune cell infiltration in pan-cancer was analyzed by the TISIDB database and showed as a heat map. Blue squares represent negative correlations, and red squares represent positive correlations. (B-F) The correlation between SH3TC2 expression and a panel of known ICMs, including CTLA4, PDCD1, CD28, CD70, VTCN1, in 367 CRC tissues was analyzed by the GEPIA database.


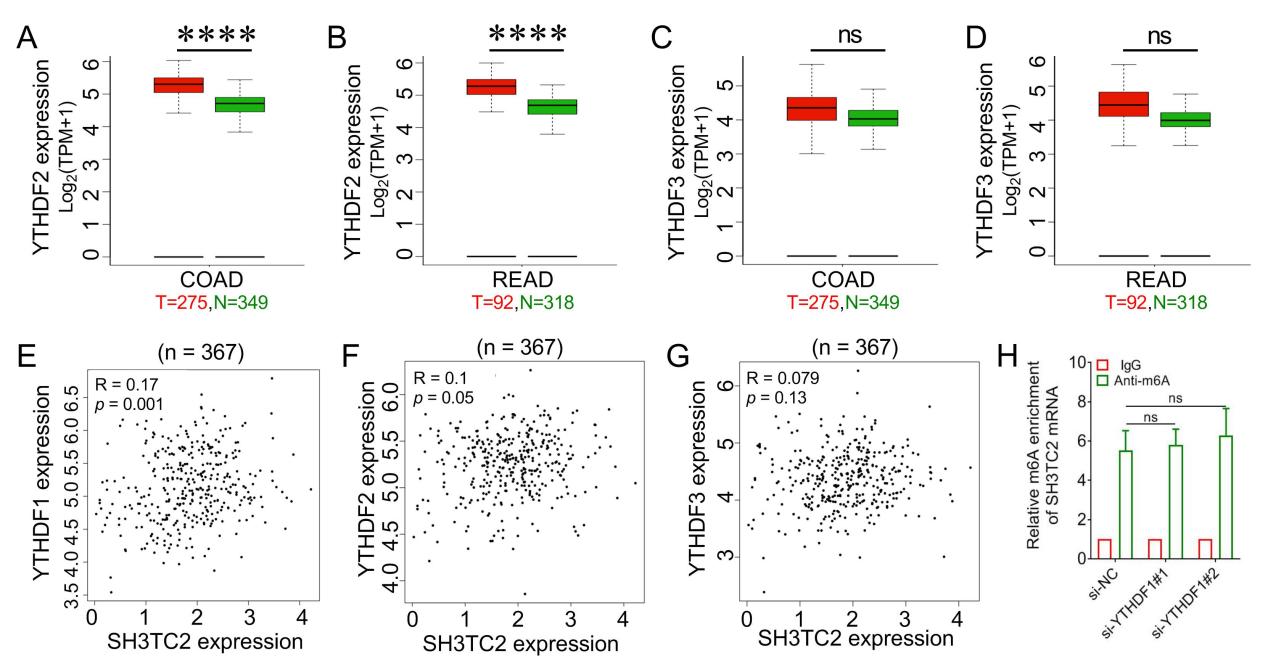


**Supplementary Figure 3. The correlation between SH3TC2 expression and YTHDF1/2/3 in CRC.** (A-B) The expression of YTHDF2 in both COAD and READ was analyzed by the GEPIA database, *****p* < 0.0001. (C-D) The expression of YTHDF3 in both COAD and READ was analyzed by the GEPIA database, ns means no significance. (E) Correlation between SH3TC2 expression and YTHDF1 in 367 CRC tissues was analyzed by the GEPIA database. (F-G) The correlation between SH3TC2 expression and YTHDF2 (F) or YTHDF3 (G) in 367 CRC tissues was analyzed by the GEPIA database. (H) MeRIP combined with qRT-PCR were applied to evaluate the m6A enrichment of SH3TC2 transcript after silencing YTHDF1 in HCT116 cells.
